# Trends in total daily dose and variability of insulin requirements in newly diagnosed children and adolescents with type 1 diabetes over 48 months

**DOI:** 10.1177/15209156251369882

**Published:** 2025-08-13

**Authors:** Chloë Royston, Julia Ware, Janet M. Allen, Malgorzata E. Wilinska, Sara Hartnell, Ajay Thankamony, Tabitha Randell, Atrayee Ghatak, Rachel E.J. Besser, Daniela Elleri, Nicola Trevelyan, Fiona M. Campbell, Roman Hovorka, Charlotte K. Boughton

**Affiliations:** 1 Institute of Metabolic Science-Metabolic Research Laboratories, University of Cambridge, Cambridge, U.K; 2 Department of Paediatrics, University of Cambridge, Cambridge, U.K; 3 Wolfson Diabetes and Endocrine Clinic, Cambridge University Hospitals National Health Service Foundation Trust, Cambridge, U.K; 4 Department of Paediatric Diabetes and Endocrinology, Nottingham Children’s Hospital, Nottingham, U.K; 5 Department of Diabetes, Alder Hey Children’s National Health Service Foundation Trust, Liverpool, U.K; 6 Department of Paediatrics, University of Oxford, Oxford, U.K; 7 National Institute for Health and Care Research Oxford Biomedical Research Centre, John Radcliffe Hospital, Oxford, U.K; 8 Department of Diabetes, Royal Hospital for Sick Children, Edinburgh, U.K; 9 Paediatric Diabetes, Southampton Children’s Hospital, Southampton, U.K; 10 Department of Paediatric Diabetes, Leeds Children’s Hospital, Leeds, U.K

**Keywords:** Type 1 diabetes, hybrid closed-loop, insulin pump, continuous glucose monitor, children, adolescents, newly-diagnosed, insulin variability

## Abstract

**Objective:**

To evaluate trends in insulin delivery and day-to-day variability of insulin requirements over 48 months of hybrid closed-loop use following diagnosis of type 1 diabetes in individuals aged 10 to 16 years.

**Methods:**

A secondary analysis of the closed-loop arm of an open-label, multicentre, randomised, parallel hybrid closed-loop trial assessing closed loop insulin delivery in newly diagnosed children and adolescents with type 1 diabetes was conducted. Mean total daily dose (TDD) over 24 hours and during the night, as well as mean total basal and bolus insulin over 24 hours, were calculated. Day-to-day variability of insulin requirements was evaluated over 24 hours and at night.

**Results:**

TDD increased from 27.2±16.1 units/day (mean±SD) at 0-3 months following diagnosis to 65.7±24.9 units/day at 42-48 months. The proportion of total daily insulin delivered as basal insulin rose from 41% to 61% over 48 months. Day-to-day variability of insulin requirements after diagnosis was high (coefficient of variation at 0-3 months: 23.3±0.9%) and remained stable over 48 months. No clinically relevant sex-based differences were observed in insulin requirements.

**Conclusions:**

During the first 48 months after diagnosis of type 1 diabetes, insulin requirements in children and adolescents more than double with hybrid closed-loop insulin delivery. Over time, a greater proportion of insulin is administered via the closed-loop algorithm, and the high day-to-day variability in insulin needs underscores the importance of initiating adaptive closed-loop systems from diagnosis.

## Introduction

Type 1 diabetes (T1D) is caused by immune-mediated destruction of insulin-producing pancreatic β-cells in genetically susceptible individuals, leading to insulin deficiency (1). The period following T1D diagnosis presents unique management challenges. Many individuals experience a partial remission, during which residual β-cell function temporarily reduces exogenous insulin requirements. However, as β-cell function declines and endogenous insulin production diminishes, insulin requirements typically increase (2). Limited research has explored the day-to-day variability in insulin requirements after diagnosis.

Hybrid closed-loop insulin delivery systems, which use glucose-responsive insulin delivery to target specific glucose levels have been shown to improve glycaemic outcomes and quality of life in people with T1D (3–7). The CLOuD randomised controlled trial showed that closed-loop insulin delivery improved glycaemic outcomes over 48 months after diagnosis compared with standard insulin therapy in children and adolescents with type 1 diabetes, but this did not appear to confer a protective effect on residual C-peptide secretion (8).

The aim of the present post-hoc analysis was to assess trends in closed-loop insulin delivery and day-to-day variability of insulin requirements in children and adolescents over 48 months following T1D diagnosis.

## Materials and methods

### Study design and population

A secondary analysis of the closed-loop arm of an open-label, multicentre, randomised, parallel hybrid closed-loop trial was conducted to assess exogenous insulin requirements in the first 48 months following T1D diagnosis. Participants aged ≥10 and <17 years were recruited within 21 days of diagnosis of T1D and were randomised to either closed-loop or standard insulin therapy for 24 months. Participants were offered a 24-month optional extension phase with the allocated treatment. The present analysis includes only participants who used closed-loop therapy. The study was approved by an independent research ethics committee.

### Closed-loop system

The Cambridge model predictive control algorithm (version 0.3.71, CamDiab Ltd, Cambridge, UK) was implemented in two hardware configurations sequentially. The FlorenceM configuration consisted of an unlocked Android smartphone running the algorithm which communicated with a modified, next-generation sensor-augmented 640G Medtronic insulin pump (Medtronic Minimed, CA, USA) through a proprietary translater, and a Medtronic continuous glucose monitor (CGM) transmitter with Guardian 3 sensor. The CamAPS FX configuration used an unlocked Android smartphone to house the CamAPS FX closed-loop app, a Dana Diabecare RS insulin pump (Sooil Development, Seoul, Korea) or YpsoPump (Ypsomed, Burgdorf, Switzerland), and either the Dexcom G6 (Dexcom, San Diego, CA, USA) or FreeStyle Libre 3 (Abbott Diabetes Care, Alameda, CA, USA) continuous glucose monitor (CGM).

Both configurations used an algorithm housed in a mobile app to automatically adjust insulin doses every 8 to 12 minutes. The algorithm was initialised by entering the user’s weight and total daily dose, while insulin sensitivity and active insulin time were automatically calculated and adjusted as necessary. The CamAPS FX algorithm adjusts insulin delivery by modulation of basal infusion rates without any need for auto-corrections, while bolus insulin delivery is user-initiated, usually for carbohydrate intake. The algorithm uses adaptive learning with respect to total daily insulin dose, diurnal variation, and insulin delivery around meals.

### Data analysis

Metrics were calculated daily for each participant and then summarised over 3-month (91-day) periods for the first 24 months and 6-month (182-day) periods for the second 24 months, starting from the day after diagnosis. All metrics were summarised with equal weight given to each participant. For inclusion in the analysis, a participant’s data was only considered if at least 50% of each 24-hour period was spent in closed-loop mode. Additionally, data from each participant was only included in a period if at least 30 days of data were available for the 91-day periods and at least 60 days for the 182-day periods.

Metrics included mean total daily dose (TDD) over 24 hours, during the day (06.00-23.59) and during the night (00.00-5.59), as well as mean basal and bolus insulin doses over 24 hours. Insulin variability was assessed by calculating the coefficient of variation (CV) of total insulin dose over 24 hours, during the day and during the night. We have previously used CV to evaluate variability of day-to day insulin delivery in other populations to understand who experiences higher variability of insulin delivery, and help inform who would benefit from more advanced therapies (12). These metrics were calculated for female and male participants, as well as for the overall group.

Data analysis was completed using R Studio (R Foundation for Statistical Computing, Vienna, Austria). Data is presented as mean ± standard deviation (SD).

## Results

A total of 48 participants (23 female and 25 male) aged 10 to 16.9 years were included in the analysis (Table 1). Due to the data inclusion criteria as outlined in the methods, the number of users in each period varied. Exact numbers for each time period are provided in Table S1. One participant was excluded as an outlier due to behavioural (deliberate carbohydrate restriction) factors impacting on insulin requirements.

### Total daily dose

**Figure 1:**
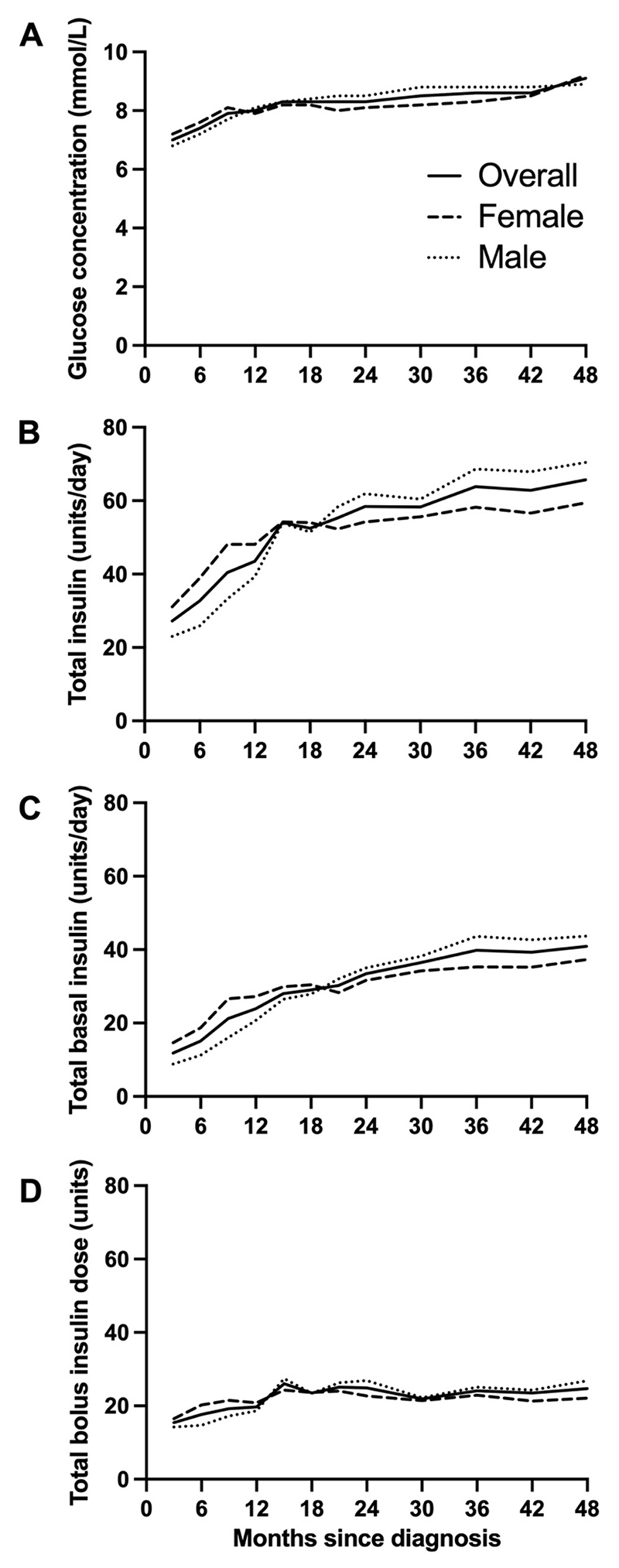
Glucose levels and insulin requirements during hybrid closed-loop insulin delivery over the 48 months following diagnosis of type 1 diabetes: (A) mean sensor glucose, (B) total daily insulin dose, (C) total daily basal insulin dose, (D) total daily bolus insulin dose.

Mean±SD TDD doubled over the first 12 months after diagnosis from 27.2±16.1 units/day at 0-3 months to 54.0±29.5 units/day at 12-15 months (Figure 1b and Table S1). Thereafter the TDD increased more slowly to 65.7±24.9 units/day at 42-48 months). A similar trend was observed with the daytime (06:00 to 23:59) TDD which increased from 24.1±14.3 units/day at 0-3 months to 47.1±26.0 units/day at 12-15 months with a slower increase to 55.5±20.4 units/day at 42-48 months (Table S1). Nighttime (00:00 to 05:59) TDD also doubled from 3.2±1.9 units/night at 0-3 months to 6.9±3.8 units/night at 12-15 months and then increased to 10.2±5.2 units/night at 42-48 months (Table S1).

We observed a trend towards lower mean TDD in females than in males for the first 18 months after diagnosis, whereas from 18 months onwards mean TDD tended to be higher in females than males although this was not tested statistically (Table S1, Figure 1b). This trend occurred in both total daytime and nighttime insulin delivery. A sensitivity analysis of TDD using higher thresholds of time in automode (>70% and >90%) showed a similar trend (data not shown).

**Figure 2:**
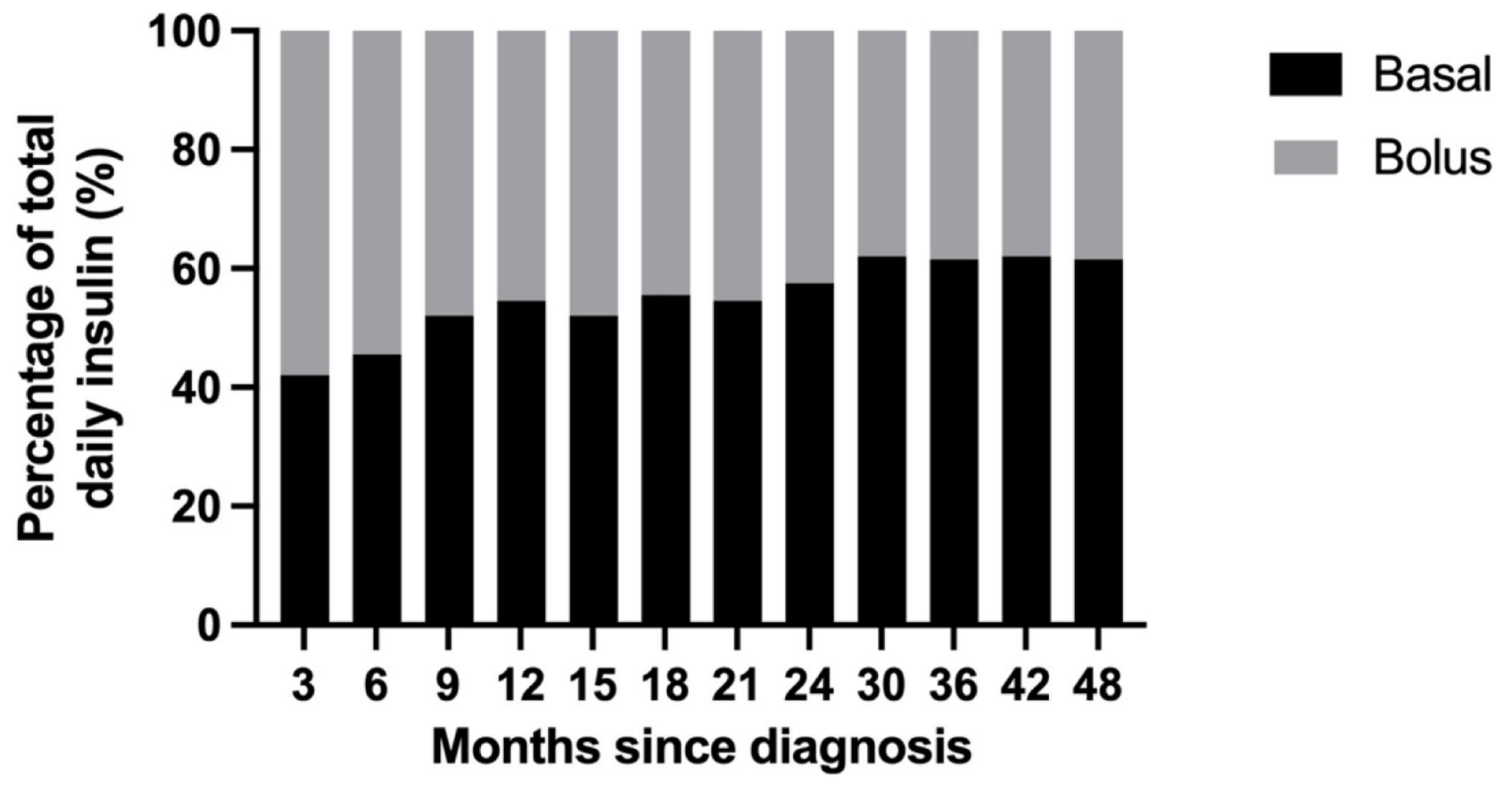
Proportion of total daily insulin as basal and bolus insulin (%) during hybrid closed-loop insulin delivery over the 48 months following diagnosis of type 1 diabetes.

Total daily basal insulin increased almost fourfold from 11.8±9.9 units/day at 0-3 months after diagnosis to 40.9±19.8 units/day at 42-48 months, while total daily bolus insulin delivery increased by less than twofold from 15.4±7.0 units/day at 0-3 months to 24.7±9.5 units/day at 42-48 months (Table S1). The proportion of TDD delivered as basal insulin increased from 41% to 61% over 48 months (Figure 2, Table S2).

### Day-to-day variability

**Figure 3:**
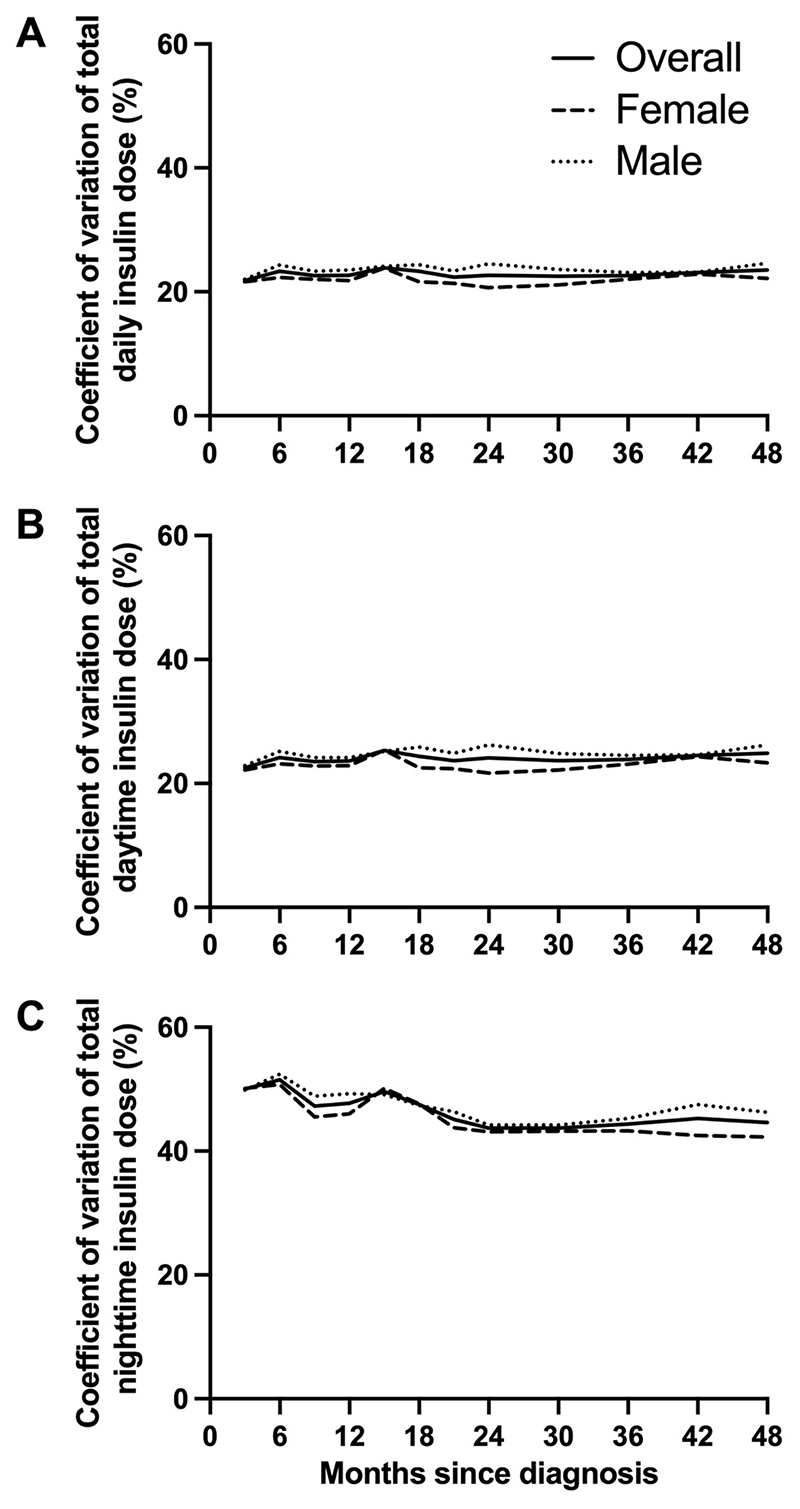
Day-to-day variability in insulin requirements during hybrid closed-loop insulin delivery over the 48 months following diagnosis of type 1 diabetes is depicted as follows: (A) overall daily variability, quantified by the coefficient of variation (CV) of total daily insulin doses (24 hours); (B) daytime variability, quantified by the CV of total insulin doses from 06:00 to 23:59; and (C) nighttime variability, quantified by the CV of total insulin doses from 00:00 to 05:59.

The CV of 24h day-to-day insulin delivery remained stable over the 48 months with the lowest CV of 21.8% at 0-3 months and highest CV of 23.9% at 12-15 months (Table S1, Figure 3a). Daytime (06:00-23:59) CV also appeared to be stable, with the lowest CV of 22.5% at 0-3 months and the highest CV of 25.3% at 12-15 months (Table S1, Figure 3b). We observed a trend towards a decrease in nighttime (00:00 to 05:59) CV from 50.0±2.0% at 0-3 months to 44.6±1.6% at 42-48 months (Table S1, Figure 3c). Nighttime CV ranged from 43.7% to 51.5% while daytime CV ranged from 22.5% to 25.3% (Table S1).

### Glucose trends

Over 48 months post-diagnosis, mean glucose increased by 2.1 mmol/L (7.0±0.8 to 9.1±1.6 mmol/L), while time-in-range (TIR, 3.9 to 10.0 mmol/L) decreased from 82.2±10.9% to 65.3±12.6% (Table S3, Figure 1a).

## Discussion

Our analysis demonstrates that total daily insulin requirements more than double with hybrid closed-loop insulin delivery over the first 48 months following a T1D diagnosis. Most of the increase in closed-loop insulin delivery occurred in the first 15 months and may relate to β-cell destruction and decreasing endogenous insulin production (8).

The CamAPS FX algorithm adjusts insulin delivery by modifying basal rates, while bolus insulin is user-initiated, primarily for meals or carbohydrate intake. Therefore, changes in basal insulin delivery directly reflect the algorithm’s adjustments. The increasing proportion of the total daily insulin composed of basal insulin over time suggests that the algorithm adapts to rising insulin requirements, while the reducing proportion of bolus insulin could be hypothesised to be due to a lack of adjustment of carbohydrate ratios or reducing bolus frequency over time. This is particularly relevant considering that missed boluses, suboptimal adherence to management and elevated diabetes distress are frequently reported in adolescent populations (9–11).

The variability in day-to-day insulin delivery, measured as CV, was relatively stable over the 48 months after diagnosis. The trend towards decreasing variability of night-to-night insulin delivery over time may reflect declining endogenous insulin secretion. The CLOuD randomised controlled trial showed that C-peptide levels declined most rapidly over the first 24 months and then decline less rapidly over the following 24 months which would support this observation (8). The variability of day-to-day insulin delivery we observed in this cohort of newly-diagnosed children and adolescents was higher (23-25%) than is reported in age-matched peers with established T1D (19%), and more comparable to the CV reported in very young children with T1D (25%) (12). Newly-diagnosed children and adolescents are therefore likely to derive particular benefit from adaptive closed-loop insulin therapy. This is also supported by the improved glycaemic outcomes in those using hybrid closed-loop system compared to those receiving standard insulin therapy reported in the original study (8).

There is growing interest in the influence of sex on the management and outcomes of T1D, with current evidence conflicting (13–15). No significant differences in glycaemic outcomes between sexes have been observed with closed-loop systems (4, 16). In the present analysis, we observed a trend towards lower closed-loop insulin delivery in females than in males for the first 18 months after diagnosis, and higher closed-loop insulin delivery in females than males thereafter. However importantly, pubertal status was not recorded in this study.

Strengths of the present analysis include the long duration of closed-loop insulin delivery following diagnosis and the use of a highly adaptive closed-loop algorithm. Limitations include the small sample size (N=48), low ethnic diversity (85% white), lack of data on pubertal status, limited participant data for some periods and the presentation of insulin as total daily dose instead of by weight as weight data was not available for all the time periods. It was also not possible to calculate how much of the total daily insulin is directly due to use of the Boost function, however the median (IQR) frequency of Boost use was 0 (0, 1) per day (data not shown).

## Conclusions

In conclusion, over the first 48 months after diagnosis of type 1 diabetes, insulin requirements in children and adolescents more than double with closed loop insulin delivery. Over time, a greater proportion of insulin is delivered by the closed-loop algorithm. The day-to-day variability of insulin requirements is high from diagnosis, supporting early use of adaptive closed loop systems after diagnosis of T1D.

## Supplementary Material

Supplementary Tables S1-S3

## Figures and Tables

**Table 1:** Demographics

Characteristics	Overall (N = 48)	Female (N = 23)	Male (N = 25)
Mean age at enrolment - years	12 ± 2	12 ± 2	12 ± 2
BMI percentile	19 ± 3	19 ± 4	19 ± 3
Race – no. (%)			
White	41 (85)	17 (74)	24 (96)
Black	1 (2)	1 (4)	0 (0)
Asian	2 (4)	2 (9)	0 (0)
More than one race	4 (8)	3 (13)	1 (4)
Glycated hemoglobin level (%)	10.7 ± 1.8	10.8 ± 2.0	10.6 ± 1.6
Diabetic ketoacidosis at diagnosis – no. (%)	15 (31)	9 (39)	6 (24)

Data presented as mean ± standard deviation
