# Supplementary material for: Trends in total daily dose and variability of insulin requirements in newly diagnosed children and adolescents with type 1 diabetes over 48 months
Source: Diabetes Technol Ther. Author manuscript; Available in PMC 2026 Jan 29. (PMC7618672; doi:10.1177/15209156251369882)
Supplement: Supplementary Tables S1-S3 [file EMS211980-supplement-Supplementary_Tables_S1_S3.pdf]

Supplementary Data

Table S1. Insulin outcomes overall and by sex

	Overall	Female	Male
**Number of included participants**			
0 - 3 months	29	15	14
3 - 6 months	38	20	18
6 - 9 months	31	15	16
9 - 12 months	35	17	18
12 - 15 months	42	19	23
15 - 18 months	41	18	23
18 - 21 months	38	19	19
21 - 24 months	41	19	22
24 - 30 months	37	16	21
30 - 36 months	39	18	21
36 - 42 months	38	17	21
42 - 48 months	37	16	21
**Total daily dose (unit/24 hrs)**			
0 - 3 months	27.2 ± 16.1	31.1 ± 19.9	23.0 ± 9.5
3 - 6 months	32.7 ± 19.8	38.9 ± 23.7	25.9 ± 11.3
6 - 9 months	40.4 ± 22.5	48.1 ± 26.4	33.2 ± 15.5
9 - 12 months	43.5 ± 21.6	48.1 ± 24.7	39.3 ± 17.8
12 - 15 months	54.0 ± 29.5	54.2 ± 30.1	53.9 ± 29.8
15 - 18 months	52.5 ± 25.7	54.0 ± 30.9	51.4 ± 21.4
18 - 21 months	55.3 ± 24.2	52.3 ± 23.9	58.3 ± 24.7
21 - 24 months	58.4 ± 25.5	54.2 ± 23.6	61.9 ± 27.0
24 - 30 months	58.3 ± 22.8	55.6 ± 23.3	60.4 ± 22.8
30 - 36 months	63.8 ± 24.6	58.2 ± 20.2	68.6 ± 27.4
36 - 42 months	62.8 ± 22.4	56.6 ± 18.9	67.9 ± 24.2
42 - 48 months	65.7 ± 24.9	59.4 ± 21.2	70.4 ± 26.8
**Total nighttime[a] dose (unit/6 hrs)**			
0 - 3 months	3.2 ± 1.9	3.5 ± 2.4	2.8 ± 1.2
3 - 6 months	4.1 ± 2.4	4.8 ± 2.8	3.3 ± 1.8
6 - 9 months	5.3 ± 3.0	6.4 ± 3.5	4.3 ± 2.2
9 - 12 months	5.9 ± 2.6	6.4 ± 2.9	5.3 ± 2.3
12 - 15 months	6.9 ± 3.8	6.7 ± 3.6	7.1 ± 4.0
15 - 18 months	7.2 ± 3.3	7.4 ± 3.7	7.0 ± 3.0
18 - 21 months	7.5 ± 3.9	6.8 ± 3.2	8.2 ± 4.4
21 - 24 months	8.3 ± 4.2	7.3 ± 3.6	9.2 ± 4.5
24 - 30 months	8.9 ± 4.4	8.2 ± 3.8	9.5 ± 4.8
30 - 36 months	10.0 ± 5.5	8.4 ± 3.4	11.3 ± 6.5
36 - 42 months	9.7 ± 4.5	8.4 ± 3.7	10.7 ± 4.9
42 - 48 months	10.2 ± 5.2	8.7 ± 3.5	11.3 ± 6.1
**Total daytime[b] dose (unit/18 hrs)**			
0 - 3 months	24.1 ± 14.3	27.7 ± 17.7	20.3 ± 8.4
3 - 6 months	28.6 ± 17.5	34.1 ± 21.1	22.6 ± 9.7
6 - 9 months	35.1 ± 19.7	41.7 ± 23.4	28.9 ± 13.4
9 - 12 months	37.7 ± 19.1	41.6 ± 22.0	34.0 ± 15.7
12 - 15 months	47.1 ± 26.0	47.5 ± 26.6	46.8 ± 26.1
15 - 18 months	45.3 ± 22.8	46.6 ± 27.6	44.4 ± 18.8
18 - 21 months	47.8 ± 20.9	45.5 ± 21.2	50.1 ± 20.9
21 - 24 months	50.1 ± 21.8	47.0 ± 20.6	52.8 ± 23.0
24 - 30 months	49.4 ± 19.1	47.4 ± 20.2	50.9 ± 18.6

30 - 36 months	53.9 ± 20.0	49.8 ± 17.7	57.3 ± 21.6
36 - 42 months	53.1 ± 18.6	48.1 ± 15.8	57.2 ± 20.1
42 - 48 months	55.5 ± 20.4	50.7 ± 18.2	59.1 ± 21.6
**Total daily basal dose (unit/24 hrs)**			
0 - 3 months	11.8 ± 9.9	14.6 ± 13.0	8.8 ± 3.3
3 - 6 months	15.1 ± 11.0	18.7 ± 13.4	11.2 ± 5.5
6 - 9 months	21.2 ± 12.6	26.6 ± 14.6	16.0 ± 7.9
9 - 12 months	23.9 ± 13.2	27.2 ± 15.7	20.7 ± 9.8
12 - 15 months	28.0 ± 16.7	29.9 ± 19.5	26.5 ± 14.2
15 - 18 months	29.0 ± 16.5	30.4 ± 18.8	27.9 ± 14.9
18 - 21 months	30.2 ± 16.9	28.3 ± 15.4	32.0 ± 18.5
21 - 24 months	33.4 ± 16.6	31.6 ± 16.2	35.0 ± 17.3
24 - 30 months	36.5 ± 17.5	34.2 ± 16.8	38.2 ± 18.2
30 - 36 months	39.8 ± 20.5	35.3 ± 15.8	43.6 ± 23.5
36 - 42 months	39.3 ± 18.9	35.2 ± 15.6	42.7 ± 21.0
42 - 48 months	40.9 ± 19.8	37.3 ± 15.5	43.7 ± 22.5
**Total daily bolus dose (unit/24 hrs)**			
0 - 3 months	15.4 ± 7.0	16.5 ± 7.3	14.2 ± 6.7
3 - 6 months	17.6 ± 10.1	20.2 ± 11.8	14.7 ± 7.0
6 - 9 months	19.2 ± 10.9	21.5 ± 13.0	17.2 ± 8.3
9 - 12 months	19.7 ± 10.2	20.8 ± 11.2	18.6 ± 9.3
12 - 15 months	26.0 ± 17.7	24.3 ± 14.7	27.4 ± 20.1
15 - 18 months	23.5 ± 14.0	23.6 ± 16.9	23.4 ± 11.5
18 - 21 months	25.1 ± 12.9	24.0 ± 13.9	26.3 ± 12.1
21 - 24 months	24.9 ± 14.9	22.7 ± 13.1	26.9 ± 16.3
24 - 30 months	21.9 ± 9.6	21.4 ± 10.6	22.2 ± 9.0
30 - 36 months	24.1 ± 10.1	22.9 ± 10.8	25.1 ± 9.7
36 - 42 months	23.5 ± 8.8	21.3 ± 8.1	25.3 ± 9.2
42 - 48 months	24.7 ± 9.5	22.1 ± 9.7	26.8 ± 9.0
**CV of day-to-day[c] insulin variability (%)**			
0 - 3 months	21.8 ± 0.8	21.6 ± 1.2	22.0 ± 1.1
3 - 6 months	23.3 ± 0.9	22.3 ± 1.2	24.3 ± 1.3
6 - 9 months	22.6 ± 0.8	22.0 ± 1.2	23.3 ± 1.1
9 - 12 months	22.7 ± 0.9	21.8 ± 1.3	23.5 ± 1.3
12 - 15 months	23.9 ± 0.8	23.8 ± 1.3	24.1 ± 1.0
15 - 18 months	23.2 ± 0.8	21.6 ± 1.1	24.4 ± 1.2
18 - 21 months	22.4 ± 1.0	21.4 ± 1.2	23.4 ± 1.6
21 - 24 months	22.7 ± 1.0	20.7 ± 1.2	24.5 ± 1.5
24 - 30 months	22.5 ± 1.0	21.1 ± 1.2	23.6 ± 1.4
30 - 36 months	22.6 ± 1.1	22.0 ± 1.5	23.1 ± 1.5
36 - 42 months	23.1 ± 1.0	22.9 ± 1.8	23.2 ± 1.1
42 - 48 months	23.5 ± 0.9	22.2 ± 1.1	24.6 ± 1.4
**CV of nighttime-to-nighttime[a] insulin variability (%)**			
0 - 3 months	50.0 ± 2.0	50.1 ± 2.9	49.9 ± 2.7
3 - 6 months	51.5 ± 2.2	50.8 ± 3.2	52.4 ± 3.1
6 - 9 months	47.3 ± 2.2	45.5 ± 2.8	48.9 ± 3.3
9 - 12 months	47.7 ± 2.3	46.0 ± 2.9	49.3 ± 3.7
12 - 15 months	49.6 ± 2.1	50.1 ± 3.9	49.1 ± 2.3
15 - 18 months	47.5 ± 2.0	47.6 ± 3.4	47.4 ± 2.4
18 - 21 months	45.1 ± 2.0	43.8 ± 2.5	46.4 ± 3.3
21 - 24 months	43.7 ± 1.6	43.1 ± 2.2	44.2 ± 2.4
24 - 30 months	43.7 ± 1.5	43.2 ± 2.0	44.2 ± 2.2

30 - 36 months	44.4 ± 1.8	43.3 ± 3.3	45.3 ± 2.0
36 - 42 months	45.3 ± 1.9	42.5 ± 2.8	47.5 ± 2.4
42 - 48 months	44.6 ± 1.6	42.3 ± 2.2	46.3 ± 2.3
**CV of daytime-to-daytime[b] insulin variability (%)**			
0 - 3 months	22.5 ± 0.9	22.2 ± 1.3	22.9 ± 1.3
3 - 6 months	24.2 ± 0.9	23.2 ± 1.2	25.2 ± 1.3
6 - 9 months	23.5 ± 0.9	22.8 ± 1.3	24.2 ± 1.2
9 - 12 months	23.6 ± 0.9	22.9 ± 1.5	24.2 ± 1.2
12 - 15 months	25.3 ± 0.9	25.4 ± 1.4	25.2 ± 1.1
15 - 18 months	24.4 ± 0.9	22.5 ± 1.2	25.9 ± 1.3
18 - 21 months	23.7 ± 1.1	22.4 ± 1.1	24.9 ± 1.8
21 - 24 months	24.1 ± 1.1	21.7 ± 1.2	26.2 ± 1.7
24 - 30 months	23.7 ± 1.0	22.2 ± 1.2	24.8 ± 1.6
30 - 36 months	23.9 ± 1.1	23.1 ± 15	24.5 ± 1.6
36 - 42 months	24.5 ± 1.1	24.3 ± 2.1	24.6 ± 1.3
42 - 48 months	24.9 ± 1.0	23.3 ± 1.1	26.2 ± 1.5

[a] Nighttime is defined as 00.00-05.59
[b] Daytime is defined as 6.00-23.59
[c] Day is defined as 00.00-23.59
Data are presented as mean ± standard deviation
CV: coefficient of variation

Table S2. Proportion of total daily dose as basal insulin

Basal insulin as percentage of total daily dose (%)	
0 - 3 months	41 ± 9
3 - 6 months	45 ± 10
6 - 9 months	52 ± 10
9 - 12 months	54 ± 11
12 - 15 months	52 ± 14
15 - 18 months	56 ± 14
18 - 21 months	54 ± 15
21 - 24 months	58 ± 14
24 - 30 months	61 ± 12
30 - 36 months	61 ± 13
36 - 42 months	61 ± 13
42 - 48 months	61 ± 12

Data are presented as mean ± standard deviation

Table S3. Glucose outcomes overall and by sex

	Overall	Female	Male
**Mean glucose (mmol/L)**			
0 - 3 months	7.0 ± 0.8	7.2 ± 1.0	6.8 ± 0.5
3 - 6 months	7.4 ± 0.8	7.6 ± 1.0	7.2 ± 0.6
6 - 9 months	7.9 ± 0.9	8.1 ± 1.0	7.7 ± 0.6
9 - 12 months	8.0 ± 0.9	7.9 ± 0.9	8.1 ± 0.9
12 - 15 months	8.3 ± 0.9	8.2 ± 0.9	8.3 ± 0.9
15 - 18 months	8.3 ± 0.9	8.2 ± 0.8	8.4 + 0.9
18 - 21 months	8.3 ± 0.7	8.0 ± 0.8	8.5 ± 0.7
21 - 24 months	8.3 ± 0.7	8.1 ± 0.7	8.5 ± 0.8
24 - 30 months	8.5 ± 0.9	8.2 ± 0.9	8.8 ± 0.9
30 - 36 months	8.6 ± 1.0	8.3 ± 1.1	8.8 + 0.9
36 - 42 months	8.6 ± 0.9	8.5 ± 1.0	8.8 ± 0.8
42 - 48 months	9.1 ± 1.6	9.2 ± 2.1	8.9 ± 0.9
**Time-in-range [3.9 - 10.0 mmol/L] (%)**			
0 - 3 months	82.2 ± 10.9	82.7 ± 9.1	81.6 ± 12.8
3 - 6 months	81.6 ± 8.7	79.8 ± 9.2	83.5 ± 8.0
6 - 9 months	74.4 ± 10.4	72.2 ± 11.8	76.9 ± 8.2
9 - 12 months	75.4 ± 10.4	77.2 ± 8.9	73.7 ± 11.6
12 - 15 months	70.3 ± 13.2	71.2 ± 12.7	69.4 ± 13.8
15 - 18 months	70.6 ± 10.1	72.1 ± 9.3	69.5 ± 10.7
18 - 21 months	70.8 ± 10.1	72.7 ± 10.4	69.2 ± 9.9
21 - 24 months	71.6 ± 8.8	73.7 ± 8.7	69.7 ± 8.7
24 - 30 months	71.2 ± 12.7	72.8 ± 12.6	69.9 ± 13.0
30 - 36 months	68.6 ± 10.4	71.3 ± 11.0	66.2 ± 9.6
36 - 42 months	68.1 ± 9.8	70.5 ± 9.7	66.0 ± 9.7
42 - 48 months	65.3 ± 12.6	66.1 ± 15.9	64.6 ± 9.6

Data are presented as mean ± standard deviation
